# The importance of moral fit to expectations of academic and professional wellbeing

**DOI:** 10.3389/fpsyg.2025.1433194

**Published:** 2025-02-26

**Authors:** Martino Ongis, David Kidd

**Affiliations:** ^1^Division of Environment, Math, Psychology and Health, Franklin University Switzerland, Lugano, Switzerland; ^2^Edmond & Lily Safra Center for Ethics, Harvard University, Cambridge, MA, United States

**Keywords:** moral fit, value congruence, wellbeing, academic expectations, moral foundations theory, ideological diversity

## Abstract

What impact does moral fit have on expectations and engagement in higher education? We conducted two studies with college and university students in the U.S. In Study 1 (*N* = 151), we manipulated the moral fit of a hypothetical student, assessing its impact on anticipated academic, social, and professional outcomes. Study 2 (*N* = 201) involved manipulating moral values within a course description to investigate whether alignment with participants’ values influenced their interest in the course and expected outcomes. Both studies demonstrate that students are aware of moral fit in higher education, associating it with more positive expectations.

## Introduction

In recent decades, the study of morality has taken center stage in social psychology, with research examining how people reason about moral situations and individual differences in moral orientations (Ellemers et al., 2019). Less attention has been given to the implications of moral reasoning for behavior in specific situations and how moral orientations shape expectations that drive behavior.

In this article we focus on higher education, examining how *moral fit* – how individual morals align with the perceived core values of a context – shapes students’ anticipated social, academic, and professional expectations. Building on propositions that people view institutions as having moral orientations and that individuals flourish when they can pursue their values, we hypothesize that students will build more positive expectations and anticipate better outcomes when their morals align with those they see as central in their academic studies. So far, little empirical research has examined directly how people seek environments aligning with their moral values. Indirect evidence, however, suggests they might. For example, people are more persuaded by arguments framed in terms of their moral orientations (Feinberg and Willer, 2015; Thomas et al., 2023), which promote fluency and feelings of authenticity, and moral fit relates to people’s decisions to live in a specific community (Chopik and Motyl, 2015). Missing from this literature, though, is evidence that people are aware of the extent of moral fit they will experience across environments, and that they anticipate better outcomes in the context of moral fit.

## Theoretical background

Our argument builds upon research showing that individuals seek environments aligned with their moral values, and that such environments promote positive outcomes. Though broader frameworks such as person-environment fit (Edwards and Shipp, 2007) and self-determination theory (Ryan and Deci, 2000) provide foundational insights into how people thrive in environments that align with their psychological needs and personal characteristics, much of the existing research has not explicitly framed these processes in terms of moral fit. However, the mechanisms underlying value congruence more generally – including its effects on belonging, motivation, and wellbeing – suggest moral fit similarly shapes students’ academic experiences. To this end, we build upon targeted theories that address value congruence while extending them to consider the potential role of moral fit in educational contexts.

One line of evidence comes from moral foundations theory (Graham et al., 2018), which proposes that moral judgments stem from five moral foundations: harm/care, fairness/reciprocity, ingroup/loyalty, authority/respect, and purity/sanctity. Because these foundations shape individuals’ perceptions of right and wrong, they might also influence preferences for institutions and environments that reflect these values. Research shows that moral foundations drive the impact of moral similarity on group identification, enhancing adherence to group norms (Schneeberger and Krupka, 2021) and fostering belonging. Supporting this idea, people are more persuaded by arguments framed in terms of their moral foundations (Feinberg and Willer, 2015; Thomas et al., 2023), suggesting that moral fit plays a role in shaping attitudes toward institutions and social groups.

A second line of evidence comes from role congruity theory, which suggests that groups receive positive evaluations when their characteristics align with stereotypical roles (Eagly and Diekman, 2005). Although this framework has been traditionally used to explain gender bias and leadership perceptions (Eagly and Karau, 2002), its core principle – that people react negatively to incongruence – may extend to moral fit. People may experience value misalignment as a form of incongruence, leading to perceptions of inauthenticity and career dissatisfaction (Bigelow et al., 2014; Tang et al., 2021). In organizations, such misalignment is linked to pay inequality and leadership ineffectiveness (Klein et al., 2021; Wang et al., 2019; Hoobler et al., 2009), highlighting how perceived incongruities between individuals and institutional values can shape important life outcomes.

A third line of research comes from research on *institutional stereotypes* (Aaker et al., 2010; Johnson and DeGarmo, 2018), specifically how individuals attribute human-like characteristics to organizations (Slaughter et al., 2004; Sassaman et al., 2019). People distinguish between organizations based on perceived traits and values (Lievens and Highhouse, 2003; Lievens et al., 2007), suggesting that institutions themselves are subject to moral evaluation. This means that perceived moral alignment, or lack thereof, can influence career and institutional choices (e.g., Lauver and Kristof-Brown, 2001; for a meta-analysis, see Kristof-Brown et al., 2005). This line of research has shown that people often abandon career aspirations despite having the skills if they perceive a mismatch between personal and organizational values.

Finally, research on value alignment and wellbeing suggests that people thrive in environments that reflect their values (Schmader and Sedikides, 2018; Aday and Schmader, 2019). When individuals feel their values are supported, they experience higher authenticity, social fit, and belonging (Walton and Cohen, 2007). Much of this work focuses on underrepresented groups, showing for instance that recognition of shared success within a group enhances belonging and psychosocial outcomes (Walton and Brady, 2021).

Overall, evidence from multiple lines of research suggests that moral fit plays a crucial role in shaping life choices and wellbeing. The ability to self-select into morally aligned environments is especially relevant in diverse societies that offer considerable opportunities for personal choice, particularly during early adulthood, a crucial period of identity development. Higher education thus offers an ideal context to explore how individuals navigate value alignment in institutional settings.

## The context of higher education

At least since Tinto’s educational persistence model (Tinto, 1982), it has been recognized that educational systems interact with student characteristics – including values – to determine one’s degree of interaction with peers and wider university systems (e.g., academic advisors). If students’ academic experiences conflict with their core beliefs and values, they may find it difficult to integrate into the university community, which can negatively impact their academic performance (Tinto, 1993). While much of this research has focused on values in general, it remains an open question whether moral value congruence, in particular, plays a role in student integration and success.

Research on values in educational contexts has mostly focused on how individuals with different personalities sort themselves into different academic departments (Vedel, 2016; Richardson et al., 2012), but recent studies have extended this to the role of value congruence in predicting outcomes. Cross-national and cross-cultural studies have consistently demonstrated that students who share their peers’ values report stronger interpersonal relationships, greater satisfaction, and improved wellbeing (Sagiv and Schwartz, 2000; Sortheix and Lönnqvist, 2015). Similarly, work on belonging has shown that uncertainty about social fit, particularly due to stigmatization, can negatively affect motivation and achievement, especially among Black students (Walton and Cohen, 2007), and that value alignment is critical for the wellbeing of underrepresented minority students (e.g., Jackson et al., 2016). For instance, minority graduate students whose doctoral work aligns with their values report greater belonging, program satisfaction, and psychosocial functioning (Miller and Orsillo, 2022).

Relatedly, research on stereotypes in educational contexts suggests perceptions of misalignment – whether in academic norms or broader institutional culture – can shape students’ choices. For example, the activation of gendered stereotypes can lead students to perceive value conflicts within certain fields. Diekman et al. (2010) found that women’s endorsement of communal goals (often associated with female gender roles) predicted lower interest in STEM, even after accounting for STEM efficacy. Similarly, Cheryan et al. (2009) demonstrated that stereotypically male, “geeky” classroom décor reduced women’s inclination to pursue computer science degrees. While such perceptions often center on social identity and career goals, they may also extend to moral values, influencing how students evaluate the moral culture of academic disciplines and institutions.

Despite these lines of evidence, there is no direct research on whether students think of academic experiences in terms of moral fit or how perceptions of moral fit shape academic expectations. At a time of heightened controversy regarding inclusivity and ideological bias in higher education (Wills et al., 2019), it is important to clarify how students respond to environments that appear to either affirm or challenge their personal moral values.

## Research overview

We conducted two studies to test whether students anticipate better academic and personal outcomes in academic environments that align with their moral values. Moral fit was operationalized using moral foundations theory. In the literature reviewed above, values are often understood in terms of Schwartz’s personal values theory (Schwartz et al., 2012), which defines values as general goals that act as organizing principles for a person or group. As such, they broadly describe what a person believes is desirable, but they may not always align with moral beliefs. For example, Sverdlik and Rechter (2020) found that religiosity moderated the extent to which participants associated personal values with being moral, and other research has demonstrated reliable links between personal values and moral concerns while affirming their status as distinct constructs (e.g., Zapko-Willmes et al., 2021). Unlike personal values, moral foundations have stronger motivational and social impacts. For example, while a person who values stimulation would probably not view someone who favored security as immoral (though perhaps a bit dull), a person who strongly values the foundation of purity would be expected to condemn someone who violated standards of chastity. Building on findings that value congruence effects are clearest for strongly held values, we propose that such effects should be observed for moral concerns. Critics argue that moral foundations overlap with established social psychological constructs like authoritarianism and social dominance orientation (Federico et al., 2013), but they do explain specific variance in ideology (Yilmaz and Saribay, 2019).

For both studies, we report all conditions and measures. Sample sizes were predetermined to achieve 85% power for detecting medium effects. The research complies with APA’s ethical standards and received approval from the Institutional Review Board of the authors’ institution. Materials and data are accessible at: https://osf.io/9zfkq/?view_only=6bad886bd1234383b51a35246b279563.

### Study 1

In this study, we tested the hypothesis that perceived moral fit predicts anticipated academic success among U.S. college students. Participants read a scenario about a college student considering graduate programs. To manipulate moral fit, we varied information about the students’ personal values and the academic discipline they were considering before participants rated moral fit and anticipated academic success.

#### Method

##### Participants

We recruited 151 participants enrolled in a U.S. college or university program through Prolific, and none were excluded. Before the study, participants provided their age, academic class standing, enrollment status, and major.

##### Instruments

Moral fit was assessed using three items (*α* = 0.92) and anticipated academic success was measured using nine items (*α* = 0.93). Both were created ad-hoc for this study.

##### Procedure

The study employed a 2×2 design with values (liberal vs. conservative) and discipline (sociology vs. business) as independent variables. Participants were randomly assigned to either a condition in which Sam, a hypothetical student, was described as concerned with care and fairness (common among political liberals) or as favoring authority (common among political conservatives; Graham et al., 2009). Next, participants were randomly assigned to read that Sam was considering a prestigious graduate program in either *sociology* or *business*. These disciplines were chosen for their perceived ideological associations in American culture, with sociology linked to liberalism and business to conservatism (Gross and Simmons, 2014; Wills et al., 2019).

The study conditions created two moral fit scenarios. In the congruent values condition, Sam’s value aligned with the perceived value of the discipline (liberal value with sociology or conservative value with business). In the incongruent values condition, Sam’s values conflicted with disciplinary values (liberal values with business or conservative values with sociology). Participants then rated moral fit and academic success.

##### Data analyses (and design)

An ANOVA tested whether moral fit and anticipated success differed between congruent and incongruent conditions. We also conducted a mediation analysis (Hayes, 2013) to examine whether moral fit mediated the relationship between condition and anticipated academic success.

#### Results

We predicted that participants would rate moral fit higher in the *congruent values* condition compared to the *incongruent values* condition. We also predicted that anticipated academic success would be rated higher in the *congruent values* condition. Finally, we aimed to investigate whether moral fit mediated the link between condition and anticipated academic success.

An ANOVA revealed that moral fit was higher in the *congruent values* condition (*M* = 3.37, SD = 1.05) than in the *incongruent values* condition (*M* = 2.87, SD = 0.96; *F*(1,149) = 8.50, *p* = 0.004, Cohen’s *d* = 0.47). Participants perceived higher moral fit when Sam was presented with liberal values and considering sociology or conservative values and considering business, compared to when Sam was presented with liberal values and considering business or conservative values and considering sociology.

As hypothesized, anticipated success ratings were significantly higher in the *congruent values* condition (*M* = 3.61, SD = 0.81) compared to the *incongruent values* condition (*M* = 3.19, SD = 0.83; *F*(1,148) = 8.85, *p* = 0.003, Cohen’s *d* = 0.48). Participants were more inclined to predict Sam’s success when they perceived alignment between their personal values and those of the academic discipline.

Next, we conducted a bootstrap analysis (Hayes, 2013) to explore moral fit as a mediator between condition and anticipated academic success. Results revealed an indirect effect of moral fit, *ß* = 0.3230, SE = 0.1123, 95% CI [0.0943, 0.5388]. The direct effect was not significant, *ß* = 0.0786, 95% CI [−0.0783, 0.2323]. Thus, the relationship between the experimental condition and anticipated academic success was fully mediated by moral fit. Participants’ perception of how well Sam’s personal values aligned with the values perceived as central in the academic discipline was the underlying factor that influenced their expectations of academic success.

#### Discussion

Study 1 found that moral fit significantly impacts academic expectations. Participants perceiving alignment between Sam’s personal values and those central to an academic discipline anticipated greater success for Sam. These findings align with past research showing that moral fit influences attitudinal outcomes (Edwards and Cable, 2009; Bao et al., 2012) and extend this research to the academic domain, adding to literature on value congruence in higher education (e.g., Sagiv and Schwartz, 2000; Miller and Orsillo, 2022).

These results must be considered alongside some limitations. The study used a hypothetical scenario, potentially limiting generalizability. In addition, comparing different academic disciplines (i.e., sociology and business) may have introduced the possibility that the results were influenced by factors that people stereotypically associate with those disciplines, but that are not directly central to our hypothesis. To address these limitations and further investigate the relationship between moral fit and academic expectations, we ran an additional study.

### Study 2

In Study 2, we tested the causal relationship between moral fit and academic outcomes by manipulating value perceptions within the context of an academic course. By controlling for discipline, we improved the validity of our study design, enabling a more direct comparison of different values on academic expectations. Unlike Study 1, participants indicated their *own* personal values instead of hypothetical student values. We predicted that expectations would depend upon the degree of moral fit.

#### Method

##### Participants

We recruited 250 U.S. college or university students via Prolific. Following pre-registered methods^1^, 34 participants were excluded (12 for missing measures, and 22 for completion times of less than 2 minutes). An additional 15 participants who gave responses with no variation on at least one of the measures used to calculate the pre-registered correlation-based measure of value congruence (e.g., entering “5” for all items) were removed. The final sample included 201 participants.

##### Instruments

Participants completed a shortened version of Part 1 of the Moral Foundations Questionnaire (MFQ; Graham et al., 2008), focusing on three moral foundations relevant in educational settings: care, fairness, and authority. The MFQ measures moral concerns by asking participants to rate the relevance of various moral considerations in their decision-making. For instance, participants are asked to rate how important it is to them that people are cared for and not harmed (care), that people are treated fairly (fairness), and that authority figures are respected (authority). Participants completed the same set of MFQ items adapted to assess the perceived centrality of care, fairness, and authority within a college course based on its course catalog description. Moral fit scores were calculated using Pearson correlation coefficients between participant’s responses on the MFQ and their ratings on equivalent items in the modified MFQ, following established methods in value congruence research (e.g., Khaptsova and Schwartz, 2016; Sagiv and Schwartz, 2000). Academic expectations were measured using a six-item scale (*α* = 0.86).

##### Procedure

Participants began by completing the MFQ addressing their own views. Next, they read a description of a business ethics course. Half received a description emphasizing care and fairness (*n* = 100), while the other half received a description emphasizing obedience to authoritative codes of ethics (*n* = 101). These descriptions, reflecting liberal and conservative values respectively, were taken from a corpus of actual course titles and descriptions (Ongis et al., 2023). Then, participants completed the manipulation check, the MFQ items adapted to assess the values embedded in the course, and the measure of academic expectations. Participants in both conditions completed a single-item measure of perceived ideological leaning of the course that served as a manipulation check, followed by the academic expectations measure. Finally, they reported demographics and political ideology.

##### Data analyses (and design)

To test the effectiveness of the manipulation, we ran *t*-tests comparing perceived importance of values between the course descriptions, as well as the single-item measure of the perceived ideological leaning of the course. To test the primary hypothesis, scores on the course evaluation measure were regressed on value congruence scores.

#### Results

Demonstrating the manipulation’s effectiveness, participants viewed the “conservative” course as prioritizing authority compared to the “liberal” course, which was seen as more liberal-leaning and giving more importance to care and fairness (see Table 1).

**Table 1:** Perceived importance of values to the “Conservative” and “Liberal” courses.

	Conservative (SD)	Liberal (SD)	*t*(199)	*p*	Cohen’s *d*
Authority	3.48 (0.82)	3.13 (0.77)	3.11	0.0021	0.44
Care	3.16 (1.00)	3.56 (1.00)	−2.78	0.0059	0.40
Fairness	4.05 (0.81)	4.36 (0.71)	−2.87	0.0045	0.41
Liberal viewpoint	4.14 (1.11)	4.72 (1.20)	−3.55	0.0005	0.50

To test our primary hypothesis, we ran a regression analysis predicting academic outcomes with moral fit. As predicted, moral fit positively predicted evaluations (*t*(199) = 5.95, *p* < 0.001, *β* = 0.38, *R*^2^ = 0.15), and this effect remained significant (*t*(198) = 5.40, *p* < 0.001, *β* = 0.34) after accounting for the absolute difference between participants’ political ideology and that they perceived as supported by the course (*t*(198) = −5.09, *p* < 0.001, *β* = −0.31). Students anticipated greater success in courses aligned with their personal moral values, independent of perceived political alignment.

We also registered two exploratory analyses to inform future research. First, we hypothesized that the difference between participants’ self-reported political ideology and their perceptions of the course’s ideological leanings would predict expectations. As expected, this difference negatively predicted positive outcomes, *t*(214) = −5.54, *p* < 0.01, *β* = 0.35, *R^2^* = 0.12. Second, we examined whether the strength of participants’ political ideology (i.e., the absolute value of the difference between self-reported ideology and the midpoint of the scale) would moderate the relationship between moral fit and expectations. After standardizing and centering each variable, we entered them along with their interaction term as predictors. The main effect of moral fit (*F*(1,197) = 36.22, *p* < 0.01, η_p_^2^ = 0.13) was qualified by a significant interaction with the strength of political ideology (*F*(1,197) = 6.58, *p* = 0.01, η_p_^2^ = 0.03), indicating that the effect of moral fit was strongest for participants with more extreme political ideology. These findings suggest the importance of further investigating political ideology’s role in perceiving and experiencing moral fit.

#### Discussion

In Study 2, we examined how moral fit influences expectations in a business ethics course. As hypothesized, participants anticipated higher interest, success, and a better overall course experience when their personal values matched the values embedded in the course description. These results replicate and extend those of Study 1, while improving design validity by controlling for discipline and comparing values within the same context. These findings underscore the importance of considering moral fit in course design and implementation.

## General discussion

Across both studies, undergraduate participants in the U.S. demonstrated awareness of moral fit in education, whether in the case of a hypothetical student or in the context of their own academic decision-making. The greater the moral fit with the academic environment, the more positive the expectations for academic and personal outcomes. These findings contribute to the literature by demonstrating that students anticipate more positive outcomes in environments that align with their moral values, extending prior research on value congruence in higher education (e.g., Sagiv and Schwartz, 2000; Miller and Orsillo, 2022). Together, our findings have important implications for psychological accounts of eudaimonic human flourishing, which draw attention to the importance of being able to life one’s life in a way that reflects one’s authentic aspirations (Ryan and Deci, 2000; Ryan et al., 2019; Sutton, 2020), as well as practical relevance to real-world efforts to improve higher education.

These studies are the first to directly examine perceived moral fit in the interaction of individuals and institutions. Previous research on moral fit and value congruence typically measured alignment between participants’ self-reported values and a target group’s reported (e.g., Chopik and Motyl, 2015) or inferred values (e.g., Sagiv and Schwartz, 2000). The present findings align with calls to integrate sociological investigations of institutions with people’s psychological experiences (Aday and Schmader, 2019), suggesting people recognize opportunities for flourishing in different institutional environments. Future research should identify specific institutional features (e.g., membership, media representations) individuals use to identify these affordances.

Understanding the role of moral fit may advance efforts to make higher education more accessible. A recent report by the National Science Foundation and National Center for Science and Engineering Statistics (NCSES) notes that while women and minority students earn degrees in science and engineering, disparities persist in subfields like computer science (NCSES, 2021). Similar patterns appear in in philosophy, where women remain underrepresented (Schwitzgebel and Jennings, 2017), and in the social sciences, where ideological diversity is limited (Honeycutt and Jussim, 2020; Horowitz et al., 2018; Wills et al., 2019). The aim is not to single out these disciplines but to emphasize that the problem of limited diversity in higher education must be understood in the context of specific academic disciplines and groups of students. When disciplines become ideologically homogeneous, they risk bias, reduced creativity, and weakened public trust in their credibility (Duarte et al., 2015). Our research suggests that acknowledging a broader range of moral values might broaden participation, though future studies should examine whether individuals would embrace moral pluralism or retreat into ideologically homogeneous environments.

These implications must be weighed against several limitations of our approach. First, the use of hypothetical scenarios in Study 1 may limit the ecological validity of our findings, as real-world academic decisions involve greater complexity and nuance. While Study 2 addressed some of this limitation by using actual course descriptions, neither study involved students making real decisions about coursework or experiencing the consequences of moral fit. In their own lives, students may prioritize financial concerns over moral fit (Fischman and Gardner, 2022), and, more broadly, the degree to which social (e.g., belonging) and non-social (e.g., inspiring work goals) benefits associated with moral fit explains its effects remains unclear. Second, our samples, recruited through Prolific, may not fully represent the diverse student populations in higher education, and future research should aim for more diverse samples to improve generalizability, both in the U.S. and other cultural contexts. Third, moral fit was tested with three, rather than five, moral foundations, limiting its generalizability to other moral and non-moral values (e.g., Sagiv and Schwartz, 2000). Future research should explicitly address the proportion of variance in outcomes attributable specifically to moral values, as opposed to broader categories of personal values. Fourth, while our findings suggest that promoting moral pluralism may enhance diversity and engagement in academic disciplines, it is important to consider potential challenges. Introducing a broader range of moral values into departments with entrenched ideological leanings could create discomfort among incumbent members, potentially reducing their sense of moral fit and engagement. Additionally, in a highly polarized society, affective polarization may lead to resistance against integrating diverse moral perspectives, as people may perceive their core values as threatened (Iyengar et al., 2019). Future research should explore how to balance conflicting values without alienating any group, perhaps through gradual integration and robust conflict resolution mechanisms (Gurin et al., 2013; De Dreu and Gelfand, 2008). Many of these limitations can be addressed in future research building on these tests of these simple but foundational hypotheses.

In conclusion, across both studies, college students displayed awareness of moral fit in higher education, and consistently expected better outcomes for students, including themselves, in academic environments aligned with their moral values. These findings suggest that considering a range of moral perspectives in academic settings could support broader student engagement across fields, effectively generating opportunities for authentic engagement for students with different moral values.

## Data Availability

The datasets presented in this study can be found in online repositories. The names of the repository/repositories and accession number(s) can be found below: https://osf.io/9zfkq/?view_only=6bad886bd1234383b51a35246b279563.

## Footnotes

^1^ https://aspredicted.org/8ZJ_XVZ
